# Dr. James Ross

**Published:** 1892-06

**Authors:** 


Dr. James Ross, one of the most prominent physicians of Toronto, died at his home in that city, April 2, 1892, aged sixty years. Dr. Ross had a large clièntele, and was especially prominent in the practice of obstetrics and diseases of children. During an active professional life of forty years he attended 6,787 cases of labor,—a remarkable number for one man to see in private practice. The deceased leaves a distinguished son, Dr. James F. W. Ross, who has already made a name for himself in the field of abdominal surgery. We offer our sympathies to the surviving members of this bereaved family.

